# Connected Health User Willingness to Share Personal Health Data: Questionnaire Study

**DOI:** 10.2196/14537

**Published:** 2019-11-27

**Authors:** Maria Karampela, Sofia Ouhbi, Minna Isomursu

**Affiliations:** 1 IT University of Copenhagen Copenhagen S Denmark; 2 United Arab Emirates University Al Ain United Arab Emirates; 3 University of Oulu Oulu Finland

**Keywords:** connected health, personal health data, data sharing, questionnaire

## Abstract

**Background:**

Connected health has created opportunities for leveraging health data to deliver preventive and personalized health care services. The increasing number of personal devices and advances in measurement technologies contribute to an exponential growth in digital health data. The practices for sharing data across the health ecosystem are evolving as there are more opportunities for using such data to deliver responsive health services.

**Objective:**

The objective of this study was to explore user attitudes toward sharing personal health data (PHD). The study was executed within the first year after the implementation of the new General Data Protection Regulation (GDPR) legal framework.

**Methods:**

The authors analyzed the results of an online questionnaire survey to explore the willingness of 8004 people using connected health services across four European countries to share their PHD and the conditions under which they would be willing to do so.

**Results:**

Our findings indicate that the majority of users are willing to share their personal PHD for scientific research (1811/8004, 22.63%). Age, education level, and occupation of the participants, in addition to the level of digitalization in their country were found to be associated with data sharing attitudes.

**Conclusions:**

Positive attitudes toward data sharing for scientific research can be perceived as an indication of trust established between users and academia. Nevertheless, the interpretation of data sharing attitudes is a complex process, related to and influenced by various factors.

## Introduction

### Background

The evolution of information and communication technologies in health care holds promise for the reformation of health care services. The development of novel technologies and their acceptance by health care providers has an impact on the design and delivery of services, resulting not only in a greater number but also better quality of services [1]. Similarly, the proliferation of sensors, either worn or in mobile devices and living spaces, contributes to the creation of a new landscape for health care services, leading the way for tailor-made interventions [2,3]. According to the International Data Corporation, 1.42 billion smartphones were sold globally in 2018 [4], and by 2020, the number of smartphone users is projected to reach 2.1 billion [5], while the number of sensors connected to the Internet of Things (IoT) is forecasted to reach between 30 and 50 billion [6]. In light of these facts, the adoption of new technologies has resulted in an increasing amount of personal data stored across various databases [7,8]. The potential benefits of using big data in health care have not only increased user expectations of more sophisticated and personalized solutions but also accelerated health care professional and practitioner efforts toward the creation of more agile connected health systems to communicate health data [9,10]. Connected health can be defined as “the collective term” regrouping “telecare, telehealth, telemedicine, mHealth, digital health, and eHealth services” [11]. It is “a comprehensive, sociotechnical model for managing health care through software solutions” [12]. Moreover, it is transforming health care services [13,14], connecting patients with caregivers and clinicians [15], and empowering them to assume greater responsibility for their own health care decisions [16]. Connected health provides individual patients, patients with chronic conditions, and users in traditionally underserved populations the potential for enhanced health care service reach at a relatively low cost; it also improves scalability, time efficiency, and tailoring and customization [17,18].

The use of personal health data (PHD) can be essential to the evolution of health care systems in general [19] and connected health systems in particular [20]. Sharing health data via connected health solutions can have a positive social impact by promoting social solidarity among patients, a positive economic impact by optimizing health care resources, and a positive environmental impact by reducing the need for transportation [21]. Connected health solutions for disease prevention, like Flu Near You [22] or HealthMap [23] that map flu symptoms and infectious disease outbreaks, are examples of the potential for commercial data sharing to prevent contagious diseases. From the perspective of patients with chronic conditions, sharing health data can be valuable in many ways. For instance, it can be used to obtain a better understanding of diagnosis and treatment options or better self-management of diseases [24,25]. Similarly, for health care professionals, potential uses are numerous, as health data can facilitate the exploring of new therapeutic treatments for life-threatening diseases like cancer [26]. Data sharing initiatives have also reinforced legal frameworks and contributed to the promotion of more transparent regulations [27-29]. From a technical point of view, the need for using health data has led to the development of interoperable connected health systems for secure data exchange [30]. Frameworks to improve privacy in cloud computing and encryption techniques to offer secure access control have been among the proposed solutions to providing reliable data sharing interventions [31,32]. Despite the potential benefits of data utilization, user willingness to share personal data is changing [33]. Therefore, understanding user attitudes is crucial for developing connected health systems in the future.

The literature that examines user behavior in terms of technology adoption and use draws on various theoretical perspectives—for example, the technology acceptance model or theory of reasoned action [32]. These theories support the arguments that users develop attitudes toward technology and there is a positive relationship between attitudes and behavioral control [32,34]. Several studies have been focusing on expanding this relationship in the context of technology [32,35]. In the context of our study, user intentions to perform actions “are assumed to capture the motivational factors that influence a behavior; they are indications of how hard people are willing to try, and of how much of an effort they are planning to exert, in order to perform the behavior” [34]. Based on this definition, a willingness to share PHD can be indicative of connected health user intention to perform an action in a given situation. In this study, the conditions under which connected health users would be willing to share their PHD were limited to a set of specific situations; thus, user willingness to share their data will be discussed within this context.

Digital technology user attitudes toward sharing different types of personal data have been examined in previous studies [33]. A study by Athey et al [36] concluded that users are willing to share personal information such as private emails in exchange for small incentives such as pizza. Pickard and Swan [37] argued that consumers, in exchange of financial rewards, would be willing to sell their anonymized health data [37]. A study by Chen et al [38] about wellness data sharing concluded that user data sharing willingness is dependent on the potential uses of the data. Interestingly, this notion is further supported when considering services like 23andMe [39] for DNA analysis and PatientsLikeMe [40] for networking. Nevertheless, willingness to share health data was found to be dependent on various parameters such as the perceived sensitivity of the information [41]. While the sharing of other types of data, related to consumption and finances for example, has been seen to entail some privacy concerns, the sharing of health data is far more complicated [42,43].

Willingness to share PHD has been found to be dependent on various other parameters. For example, users are motivated to share their PHD in exchange for care improvements, better public health [44], or health information exchange [45]. Pickard and Swan’s study [37] concluded that attitudes toward data sharing for scientific purposes are becoming more positive. Additionally, users make a distinction between deidentified data and data with personally identifiable information. In a study by Weng et al [46], the majority of the respondents (89%) stated that sharing anonymized clinical data for research is preferable to sharing identifiable data. These studies confirm the idea that users desire to have control over their data sharing preferences. Individual control over data sharing preferences and patient-centered sharing models have been indicated to have positive effects on user data sharing willingness [47,48]. For example, 59% of the participants in a study by Weitzman et al [49] stated that they would prefer an opt-in data sharing model.

Apart from control over data sharing preferences, knowledge and trust have been found to contribute to more positive attitudes. Previous findings support the argument that it is unimportant for participants to fully comprehend different types of consent; nevertheless an opt-in model of informed consent is valued as a more trusted data sharing practice [50,51]. The relationship between consent and trust is fragile. Providing consent to share data cannot be equated to having trust [52]. When individuals consider sharing health information, they often weigh the personal benefits against potential risks [53-55]. Concerns of health information privacy are found to be a common barrier in sharing [56-60]. In this study, user willingness to share PHD is concerned with attitudes toward sharing health data. Data that are available online are indicative of different facets of people’s lives and can be informative about their health [8,61]. These digital traces of everyday life are the product of users’ active or passive interaction with the network [62]. Therefore, in the context of this study, PHD refers to the available online information that is the product of users’ active or passive interaction with the network and is indicative of their health. Data sharing attitudes have been explored in previous research. Nevertheless, the implementation of the new General Data Protection Regulation (GDPR) less than a year ago has led to renewed interest in this topic.

### Objectives

Over the years, researchers have been paying attention to people’s willingness to share their PHD. As technology and legal frameworks evolve, user attitudes are also undergoing a shift. This study explores and discusses current attitudes across four European countries and addresses the following research questions (RQs):

RQ1: Are connected health users willing to share their PHD?

RQ2: Under what conditions are they are willing to share their PHD?

In order to interpret the results of the study, the RQs will be discussed along the following parameters: participant age, area of residence, country of residence, education, and occupational group.

## Methods

### Research Design

The purpose of this study was to investigate user willingness and conditions for sharing their PHD. This paper presents findings from a household questionnaire survey designed by Sitra Innovation Fund and distributed by Kantar TNS Oy, a global market research and information group, in December 2018. Sitra is a Finnish Innovation Fund that through its research aims to influence European policy makers toward more sustainable well-being on social, financial, and ecological levels. The data used in this study are a subset of data from large-scale research conducted within the framework of Sitra’s IHAN project [63]. The aim of the IHAN project is twofold: to develop foundations for a fair and human-driven data economy by creating a method for data exchange and influence regulatory development toward fair use of data through European Union (EU) policy makers.

In regard to the research design of the survey, author MI has been actively involved in its development as a scientific advisor. The content of the survey was designed by Sitra through collaborative team processes to get an overall understanding of the data-driven attitudes after the implementation of the new GDPR. The website survey design was developed by Kantar TNS Oy in collaboration with Sitra. Quantitative surveys have been used by prior studies to explore and discuss user perspectives [64-66]. A survey questionnaire was chosen as research design because our goal was to get an understanding of how European countries are in regard to data economy–related topics. More specifically, we aimed to present an overview of the current landscape pertinent to user willingness and conditions for sharing their PHD after the implementation of the new GDPR. Aligned with this aim, the survey presented here was one of the first steps in a multiyear pilot project administered by Sitra. A quantitative method was chosen by Sitra to give an overview of data economy–related topics from the citizen’s point of view, and later steps of the IHAN project will investigate the findings highlighted by the survey with more qualitative approaches.

### Survey Questions

The original online survey comprised 27 questions, both open- and close-ended, to collect data relevant to background characteristics of respondents, attitudes toward services, and trust toward services and data management. For the purpose of this paper, none of the open-ended questions were used, and we only considered two of the closed-ended questions. The questions we chose to include were close-ended, as the goal was to get a quantitative overview of attitudes. The two questions measured user willingness to share and conditions of sharing their PHD.

RQ1: Are connected health users willing to share their PHD?

RQ2: Under what conditions are they are willing to share their PHD?

Data sharing in this context was pertinent to various purposes such as scientific research, public interest, and in exchange for services or financial benefits. Additionally, several sociodemographic background questions were administered to the participants to capture factors that might influence user willingness. The questionnaire was translated by professional translators from Finnish to the official language of each country. For the purpose of analysis and reporting, the questions and related responses were translated back to Finnish and English to ensure a common understanding. The survey was conducted anonymously in compliance with the EU GDPR legal framework.

### Data Collection

Four European countries were chosen, as GDPR is an EU regulation, even though it has global implications. Data collection was carried out from December 6 to 18, 2018, in Finland, the Netherlands, Germany, and France. The inclusion criteria were consent for participation in the survey and a self-declaration of being at least 18 years of age. Random sampling was performed by Kantar TNC Oy and was representative of the age, gender, and locality. Participants were invited to participate in the survey via phone or email communication. The questionnaire was delivered in the official language of each country. All participants were given financial compensation upon the completion of the online survey (Multimedia Appendix 1). The average completion time of the online survey was 12 minutes. Web-based sampling has been seen to be an effective medium to recruit participants, as it enables remote access and complies with individual preferences [67]. To comply with the emerging requirements of quality data collection in online surveys, we report our results based on the Checklist for Reporting Results of Internet E-Surveys (CHERRIES) [68].

### Data Analysis

Analysis of the raw dataset was performed by Kantar TNS Oy using SPSS Statistics (IBM Corp) software. In this study, we used the descriptive statistics that we were given by Sitra and Kantar TNS Oy to generate visualizations. The data visualizations were used for the interpretation of the results.

## Results

### Overview

The online survey included a total of 8004 respondents: 2000 from Finland, 2004 from Germany, 2000 from the Netherlands, and 2000 from France. The sample was representative in terms of gender, age, and locality. The completion rates were 84.32% (2000/2372) in Finland, 53.27% (2004/3762) in Germany, 48.77% (2000/4101) in the Netherlands, and 54.10% (2000/3697) in France.

### Demographics

Table 1 presents an overview of the participant demographics (values show the average of the percentage of all four countries). Among the participants, 49.00% (3922/8004) were male, 50.00% (4002/8004) were female, and 1.00% (80/8004) did not indicate their gender. The age distribution of the participants was between 18 and 65 years. Nearly a quarter (2001/8004, 25.00%) of the participants received compulsory education, 14.01% (1121/8004) received academic education, 58.00% (4642/8004) received other types of education, and 3.00% (240/8004) did not specify their education. Participants were also asked to specify the occupational group to which they belong, and 17.00% (1361/8004) were in a managerial position, 10.99% (880/8004) were in junior positions, 27.01% (2162/8004) were workers, 6.00% (480/8004) were self-employed or sole traders, 6.00% (480/8004) were students, 11.99% (960/8004) were pensioners, and 3.00% (240/8004) replied “don’t know.” The remaining 18.00% (1441/8004) of the participants did not fall within one of the previous categories. As for the types of living quarters, 40.00% (3202/8004) of them lived in a city, 34.00% (2721/8004) in a town or an urban area, 22.00% (1761/8004) in the countryside, and 4.00% (320/8004) did not know. See Multimedia Appendix 2 for the questionnaire survey results.

**Table 1:** Background information of respondents (N=8004).

Characteristics		Value^a^, n (%)	
**Gender**			
	Male		3922 (49.00)
	Female		4002 (50.00)
	Other		80 (1.00)
**Age in years**			
	18-34		2561 (32.00)
	35-44		1521 (19.00)
	45-65		3922 (49.00)
**Region type**			
	City		3202 (40.00)
	Town/urban area		2721 (34.00)
	Countryside		1761 (22.00)
	Do not know		320 (4.00)
**Education**			
	Compulsory education		2001 (25.00)
	Academic education		1121 (14.01)
	Other^b^		4642 (58.00)
	Do not know		240 (3.00)
**Occupational group or status**			
	At school or student		480 (6.00)
	Worker		2162 (27.01)
	Self-employed or sole trader		480 (6.00)
	Junior white collar		880 (10.99)
	Managerial position/senior		1361 (17.00)
	Pensioner		960 (11.99)
	Other^c^		1441 (18.00)
	Do not know		240 (3.00)

^a^Average of the percentage for all four countries.

^b^Other education corresponds to vocational education, matriculation, or other types of education.

^c^Other occupation corresponds to other types of jobs or status such as at-home mother/father.

### Research Question Results

**Figure 1:**
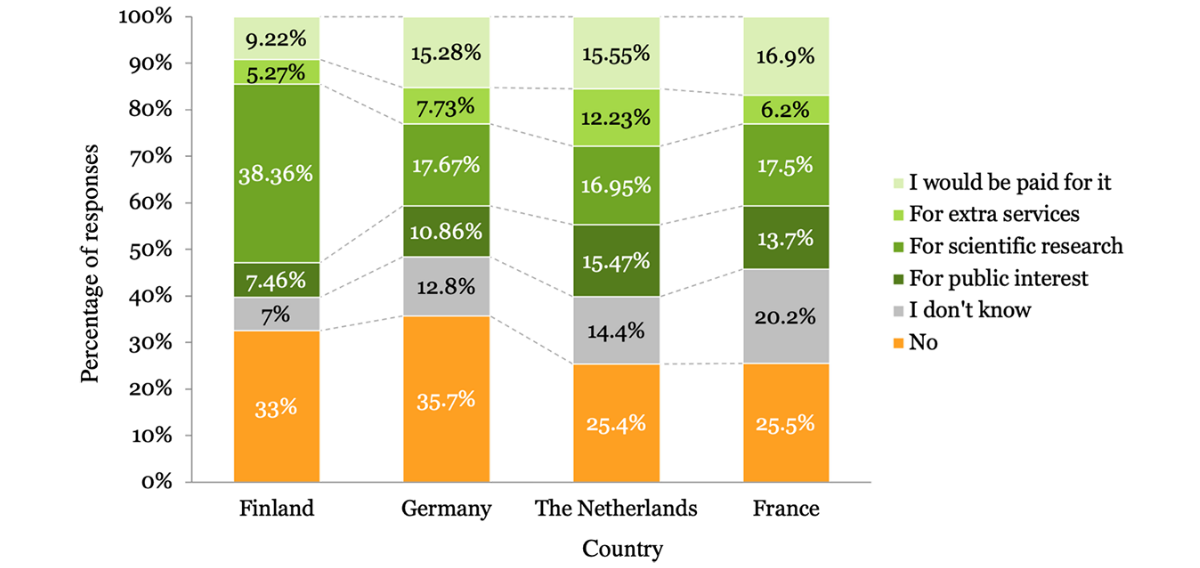
Results per country.

**Figure 2:**
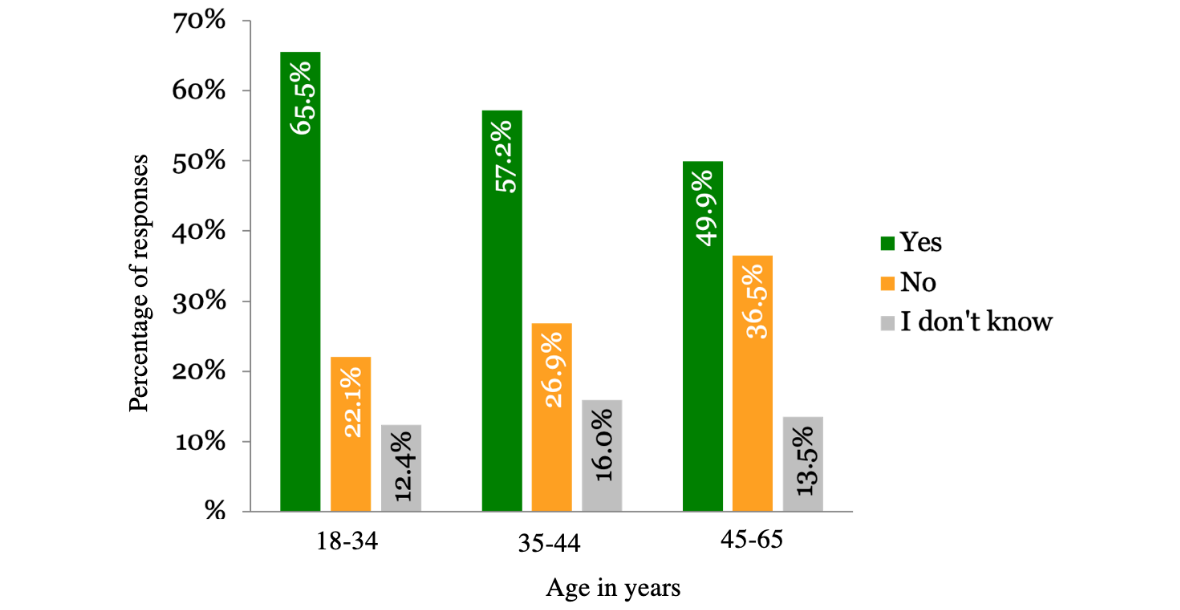
Results per age category.

**Figure 3:**
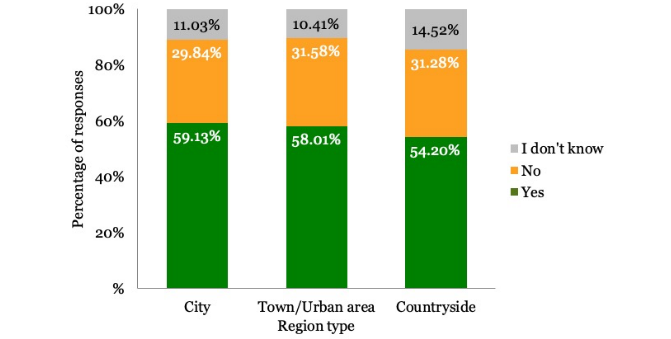
Results per region type.

**Figure 4:**
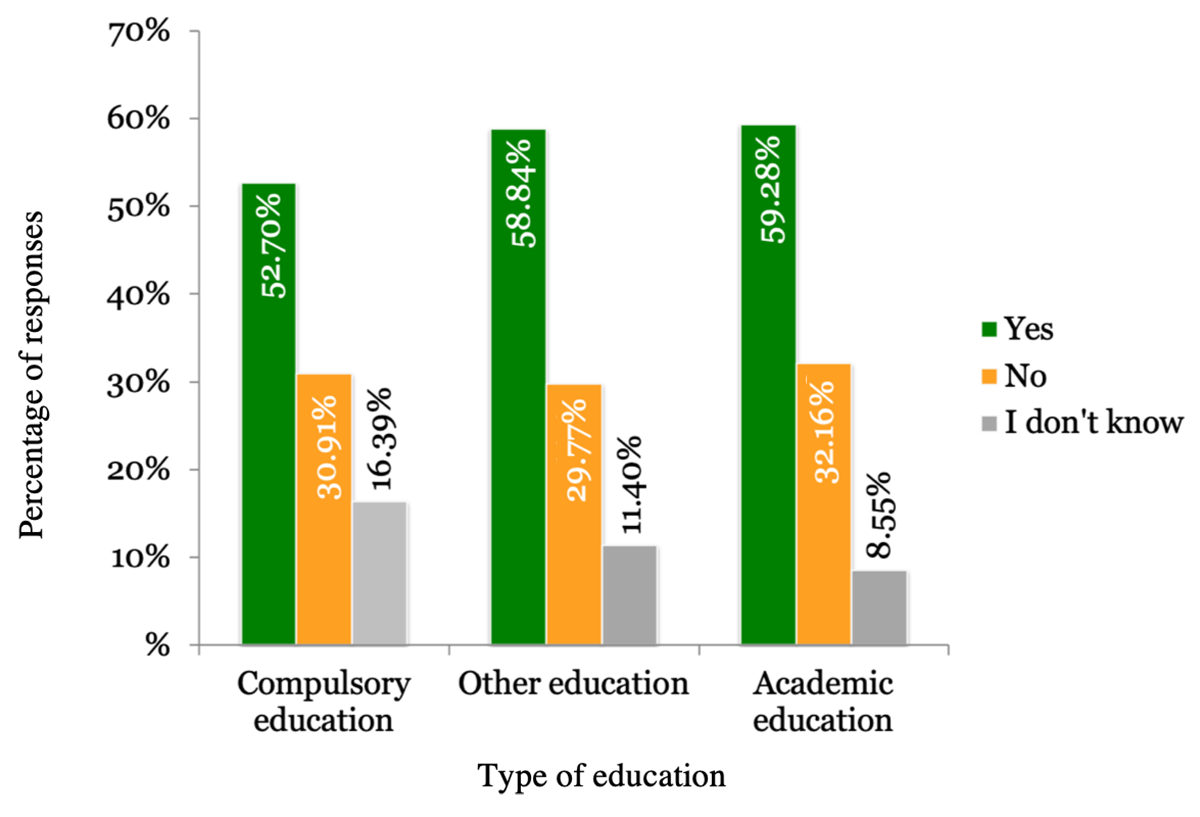
Results per education level.

**Figure 5:**
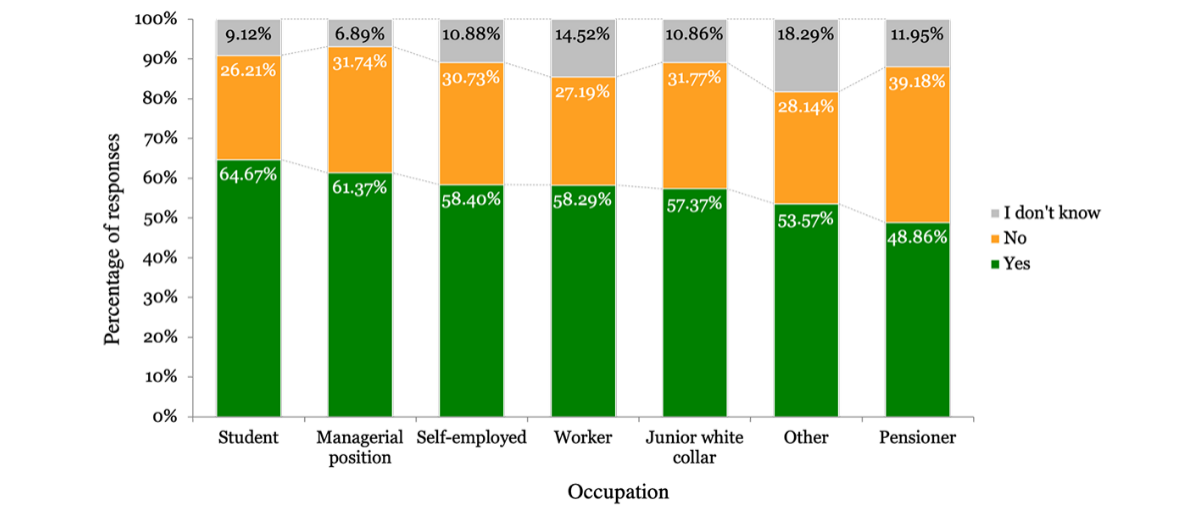
Results per occupational position type.

The majority of respondents were willing to share their PHD under specific conditions as shown in Table 2. The results differed according to the country the respondents were from. Figure 1 shows the country-wise distribution of the participant responses. Gender of the participants did not impact the results, although men (2234/3922, 56.96%) were slightly more willing to share their health data compared with women (2239/4002, 55.95%). Regarding age, young people were more willing to share their data than older people, as shown in Figure 2. Figure 3 shows that participants living in cities and urban areas were more willing to share their PHD compared with those living in the countryside. Figure 4 presents the results per education level of participants. Figure 5 presents the results according to the respondent occupation type.

**Table 2:** Participant responses about the conditions of sharing their personal health data (N=8004).

Responses	Value^a^, n (%)
No	2384 (29.78)
Information is used for scientific research	1811 (22.63)
I would be paid for it	1139 (14.23)
I don’t know	1092 (13.64)
Data is used for purposes of public interest	949 (11.86)
I would be offered extra services or individual service	628 (7.85)

^a^Average of the percentage for all four countries.

## Discussion

### Principal Findings

The aim of this study was to explore user willingness to share their digital PHD across four European countries. The four countries were chosen to represent countries in the EU that have a medium or high level of digitalization and availability of digital health data. Our findings show that the majority of users are willing to share their health data (RQ1), most often preferring to share it for scientific research (1811/8004, 22.63%; RQ2). User willingness was not strongly gender-related; however, our findings suggest that age, education, and occupation were prevalent factors affecting user attitudes.

In line with previous literature, our findings indicate that users are willing to share their PHD for research purposes [37,69]. This study extends previous research by providing deeper insights into the factors that shape user attitudes. The factors found to be affecting willingness were age, educational attainment, and occupational status. We found a negative relation between age and willingness; as users grow older, they tend to be more reluctant about data sharing, with pensioners having the highest percentage of negative responses (376/960, 39.17%). Older age has been connected with a lack of knowledge over factual risks and growing concerns over possible data misuse and security breaches [53,70]. Aging has been associated with the development of health conditions. Taking that into consideration along with the aversion of older people to sharing their health data due to privacy concerns can further support our results. Projections about the world population predict that in the future the older population will surpass younger. This change can introduce further implications in regards to data-sharing reluctance. Age has also been associated with digital literacy and competency in using digital devices [71,72]. Digital competency influences the way users interact with services [73] and is age-dependent in the sense that users grow up accustomed to different technologies. This means that they have different mental models regarding technology and the way it works [74,75]. In order to keep up with digitalization trends, users need to develop new competences or evolve their existing competences [76]. In regard to digital literacy, user willingness to exchange and use digital health information also decreases as they grow older, and the same trend applies to the willingness to learn to use online health technologies [71]. Education and occupation are also factors that influence user willingness. Lower levels of education are associated with lesser willingness to share data, possibly due to higher exposure to media and the misuse of information [54]. As for the occupational groups, pensioners were found to be the most reluctant to share PHD. Considering the similarity of trends in education and occupation, we could argue that higher educational attainment often leads to occupation at top-level or management positions, which has a positive impact on willingness to share PHD.

Current user trends in the four European countries were also informative about the impact of digitalization of health care services. The four countries that participated in the online survey belong to different groups in relation to the use of internet services. Finland and the Netherlands have the most advanced digital economies, while Germany and France belong to the medium performing group, with France ranking in the lowest position [77]. As for the level of digitalization in health care in EU, reports suggest that eHealth is still in its infancy. According to the Digital Economy and Society Index for Digital Public Services, less than 1 in 5 EU citizens have used eHealth services in the previous year [77]. Based on our results, users from countries that have medium performing digital economies were found to be more unwilling or unprepared to share their PHD. Compared with other European users, German users were the most reluctant to share their PHD, while more French users did not know if they would be willing to do so. In contrast, the Finnish participants, despite having the second highest number of negative responses toward willingness to share health data, had the highest percentage of willingness to share data for scientific research. The Dutch participants were the most willing to share their data. These findings likely correspond to the advanced levels of digitalization in Finnish and Dutch economies. However, culture-specific differences between the four countries could also play a role in the interpretation of the results. For instance, it has been reported in the past that German are apprehensive about experiencing negative consequences as result of privacy violations [72].

In regard to the conditions under which users would be willing to share their PHD, scientific research and financial incentives were prevalent. This is consistent with previous findings [45,72,77]. Finnish users had the most positive attitudes toward sharing for scientific research, which is not surprising as Finland has the highest percentage of users of eHealth services [77], while in terms of the state of open government data publication, Finland ranks fifth and France fourth [78]. Reflecting upon this, we could argue that in both countries data are valued as fuel for research. France has a long history of strict data protection [79], legal flaws, vagueness, and data mishandling, as well as political instability, which might influence citizen attitudes toward data sharing [80]. Dutch users reported the highest willingness to share PHD for public interest. The case of the Netherlands is particular due to the fact that since 2012, the country has imposed stricter legislation for ePrivacy, requiring website users to have active control over their personal information online [81]. This may explain positive user attitudes toward public service providers.

While sharing for research purposes was the most prevalent condition, a disregard of public entities may be perceived as an indicator of trust issues. Incidents of data breaches in public health care are numerous [82], which may have a negative impact on users’ data sharing attitudes. In contrast to Pickard and Swan’s [37] study, many of our users responded that personalization of services is not a strong incentive for them to share their PHD [83]. Consistent with previous studies [44], older participants where less motivated to share their health data in exchange for financial benefits. As for education and occupation, those with higher qualifications preferred to share data for research purposes. In other words, higher educational attainment has a positive relation with willingness to share for scientific research. Although we do not have information about the health status of participants, an interesting aspect to consider is that patients with chronic or terminal diseases have more positive attitudes toward data sharing for scientific research [37,46,84]. Sharing data for research purposes has been related to altruism. From a cognitive standpoint, emotions may also play an essential role in data sharing willingness. In general, the role of emotions in decision making, specifically trust and concern, has been seen to have an effect on user behaviors [54].

Willingness toward sharing PHD is related with privacy and security considerations. The privacy calculus theory suggests that risk factors, such as the purpose for which the shared information will be used, are related to user willingness [55]. According to the theory, understanding user attitudes is a complicated task. Some researchers have claimed that stricter controls in privacy settings would increase user willingness [85], while others argue that factors such as social influence can lead to similar results [86]. Deidentification of data and an opt-in model of informed consent are also solutions that have been seen to have positive effects on data sharing attitudes [46,50,87]. Recent studies on secondary use of data have indicated that users are generally willing to accept the sharing of their health data for research purposes, without explicit consent [88]. User control over accessing and sharing PHD is reflected throughout previous research for centralizing patient data, and, therefore, future interoperable health care interventions should be aware of these attitudes [60]. Technology competency and the type of information to share are also connected to privacy concerns [37,89]. Nevertheless, what cannot be neglected is that technology itself cannot handle upcoming challenges. A more holistic approach should also include proactive legal and ethical guidelines in order to increase user data sharing willingness as well as secure data exchange between health care systems in the future.

### Limitations

The interpretation of data sharing attitudes is a complex process related to and influenced by various factors [84] including the historical or regulatory context of data sharing in each nation [81]. In this study, we aimed to explore and present an overview of the data sharing perspectives adopting a quantitative approach. The research approach of this paper is related to the scope of the IHAN project and aims to present an overview of user data sharing attitudes in four European countries; therefore, the generalization of results is limited in this scope. We suggest that future qualitative or mixed methods approaches could articulate, distill, and contextualize knowledge in relation to the causality of user willingness toward sharing PHD. The analysis and discussion of the results in this study relied only on descriptive statistics and visualizations. Combined with the quantitative nature of the research, it minimizes threats to the validity of the conclusions. The inclusion of a representative sample in relation to gender, age, and locality also mitigates the risk of sampling bias. In addition, the bias related to sampling is minimum due to the random selection of the study participants. The high response rates can probably be attributed to the financial compensations that participants have received [90]. A methodological limitation, which is common in Web-based surveys, concerns the participation rate and atypical timestamp [91]. The lack of information about these two metrics raises implications about whether the participants are representative of the population intended to be analyzed and about the quality of responses in relation to the time elapsed between the dispatch of the survey and its completion. Another consideration regards a limitation that can arise from the translation and back-translation of the questionnaire and results. Although no pilot testing has been performed to validate the translated items, the development of the questionnaire by specialists, use of a consistent team of professional translators throughout the project, and quantitative nature of the study has minimized the risk.

### Conclusions

Our findings reinforce the idea that the majority of connected health users are willing to share their PHD and prefer to do so for scientific research. Furthermore, this study highlights that age, education, and occupation as well as the level of digitalization in the country are significant factors affecting user data sharing attitudes. Positive attitudes toward scientific research highlighted by our results can be perceived as an indication of trusted relations between users and academia.

Our results have both theoretical and practical implications. The enforcement of the new GDPR in Europe demands more active participation of users in the management of their personal data. Nevertheless, this requires users to be knowledgeable and fully comprehend their rights and options over privacy settings. Educating users on the benefits and safety of data sharing could facilitate fair handling of data and increase trust. From the perspective of service providers, future efforts need to be directed toward simplification of privacy statements and reconsideration of systems design. The provision of user-friendly interfaces to enable faster and seamless screening of privacy settings could be another practical implication for technology developers. From the standpoint of policy makers, this study can facilitate shifts in policies toward accommodating user needs more effectively. Educating users, information technology professionals, and service providers on the importance of implementing public policy that supports responsibility of user-mediators while identifying gaps in policy frameworks are some of the steps that stakeholders should consider in the creation of future health care systems.

## Appendix

Multimedia Appendix 1 Completed Checklist for Reporting Results of Internet E-Surveys.

Multimedia Appendix 2 Questionnaire survey results.

## Abbreviations

CHERRIES: Checklist for Reporting Results of Internet E-Surveys
EU: European Union
GDPR: General Data Protection Regulation
IoT: Internet of Things
PHD: personal health data
RQ: research question
