# Supplementary material for: Connected Health User Willingness to Share Personal Health Data: Questionnaire Study
Source: J Med Internet Res. 2019 Nov 27;21(11):e14537. doi: 10.2196/14537 (PMC6906622; doi:10.2196/14537)
Supplement: Multimedia Appendix 1 [file jmir_v21i11e14537_app1.docx]

Multimedia Appendix 1. CHERRIES checklist.

| *Item category* | *Checklist item* | *Survey compliance* |
| --- | --- | --- |
| *Design* | Describe survey design | Population 18-65, quotas on age, gender and area |
| *^a^ IRB approval and informed consent process* | IRB approval | Consent given when joined the online panel |
|  | Informed consent | Yes |
|  | Data protection | Compliant with the new GDPR |
| *Development and pre-testing* | Development and testing | Electronic questionnaire, open only for randomly selected participants |
| *Recruitment process and description of the sample having access to the questionnaire* | Open survey versus closed survey | Open and Closed |
|  | Contact mode | Recruited to the survey, either by phone or online |
|  | Advertising the survey | No |
| *Survey administration* | Web/E-mail | Participants were sent an invitation with a link to the survey |
|  | Context | - |
|  | Mandatory/voluntary | Voluntary |
|  | Incentives | 100 points for an online shop for the young, 15 points for the older (one point = 0,01€) *^b^ FI*  27 cents for completes/ 5 cent for screener only *^b^ GER , ^b^ NL,  ^b^FRA* |
|  | Time/Date | December 6 to 18, 2018 |
|  | Randomization of items or questionnaires | All the questions in the same order, but in matrix questions the statements were rotated |
|  | Adaptive questioning | - |
|  | Number of Items | One question/statement per screen |
|  | Number of screens (pages) | 27 |
|  | Completeness check | Yes |
|  | Review step | No |
| *Response rates* | Unique site visitor | After completing the survey the link can no longer be accessed |
|  | ^c^ View rate | *FI* 2372, *GER* 3762, *NL* 4101, *FRA* 3697 |
|  | ^d^ Participation rate | - |
|  | ^e^ Completion rate | *FI* 84%, *GER* 53%, *NL* 48%, *FRA* 54% |
| *Preventing multiple entries from the same individual* | Cookies used | No |
|  | IP check | Yes |
|  | Log file analysis | - |
|  | Registration | Participants register to the panel platform |
| *Analysis* | Handling of incomplete questionnaires | Marked as incomplete and not used |
|  | Questionnaires submitted with an atypical timestamp | - |
|  | Statistical correction | Weighting |

^a^ IRB: Institutional Review Board

^b^ FI: Finland, GER: Germany, NL: the Netherlands, FRA: France

^c^ Ratio of unique survey visitors/unique site visitors

^d^ Ratio of unique visitors who agreed to participate/unique first survey page visitors

^e^ Ratio of users who finished the survey/users who agreed to participate
