# Supplementary material for: Connected Health User Willingness to Share Personal Health Data: Questionnaire Study
Source: J Med Internet Res. 2019 Nov 27;21(11):e14537. doi: 10.2196/14537 (PMC6906622; doi:10.2196/14537)
Supplement: Multimedia Appendix 2 [file jmir_v21i11e14537_app2.docx]

The questionnaire’s results per category.

| *Category* | *No* | *I don't know* | *For public interest* | *For scientific research* | *For extra services or individual service* | *I would be paid for it* |
| --- | --- | --- | --- | --- | --- | --- |
| Total | 29.78  (2384/8004) | 13.64  (1092/8004) | 11.86  (949/8004) | 22.63  (1811/8004) | 7.85  (628/8004) | 14.23  (1139/8004) |
| Finland | 32.54  (651/2000) | 7.15  (143/2000) | 7.46  (149/2000) | 38.36  (767/2000) | 5.27  (105/2000) | 9.22  (184/2000) |
| Germany | 35.67  (715/2004) | 12.79  (256/2004) | 10.86  (218/2004) | 17.67  (354/2004) | 7.73  (155/2004) | 15.28  (306/2004) |
| The Netherlands | 25.37  (507/2000) | 14.45  (289/2000) | 15.47  (309/2000) | 16.95  (339/2000) | 12.23  (245/2000) | 15.55  (311/2000) |
| France | 25.54  (511/2000) | 20.16  (403/2000) | 13.67  (273/2000) | 17.54  (351/2000) | 6.20  (124/2000) | 16.89  (338/2000) |
|  |  |  |  |  |  |  |
| Male | 30.41  (1193/3922) | 12.63  (495/3922) | 11.46  (449/3922) | 20.97  (822/3922) | 8.69  (341/3922) | 15.85  (622/3922) |
| Female | 29.68  (1188/4002) | 14.37  (575/4002) | 12.37  (495/4002) | 24.47  (979/4002) | 6.61  (265/4002) | 12.50  (500/4002) |
|  |  |  |  |  |  |  |
| 18-34 y | 22.06  (565/2561) | 12.40  (318/2561) | 12.89  (330/2561) | 25.27  (647/561) | 11.17  (286/2561) | 16.22  (415/2561) |
| 35-44 y | 26.86  (408/1521) | 15.96  (243/1521) | 10.35  (157/1521) | 21.58  (328/1521) | 8.89  (135/1521) | 16.35  (249/1521) |
| 45-65 y | 36.55  (1433/3922) | 13.53  (531/3922) | 11.77  (462/3922) | 21.19  (831/3922) | 5.04  (198/3922) | 11.92  (467/3922) |
|  |  |  |  |  |  |  |
| City | 29.84  (955/3202) | 11.03  (353/3202) | 14.05  (450/3202) | 24.26  (777/3202) | 6.89  (221/3202) | 13.93  (446/3202) |
| Town/Urban area | 31.58  (859/2721) | 10.41  (283/2721) | 11.17  (304/2721) | 24.99  (680/2721) | 7.96  (217/2721) | 13.90  (378/2721) |
| Countryside | 31.28  (551/1761) | 14.52  (256/1761) | 10.83  (191/1761) | 19.39  (341/1761) | 8.63  (152/1761) | 15.35  (270/1761) |
|  |  |  |  |  |  |  |
| Compulsory education | 30.91  (619/2001) | 16.39  (328/2001) | 12.01  (240/2001) | 19.55  (391/2001) | 6.63  (133/2001) | 14.50  (290/2001) |
| Academic education | 32.16  (360/1121) | 8.55  (96/1121) | 12.70  (142/1121) | 26.78  (300/1121) | 7.35  (82/1121) | 12.45  (140/1121) |
| ^a^Other education | 29.77  (1382/4642) | 11.40  (529/4642) | 12.08  (561/4642) | 23.86  (1108/4642) | 8.25  (383/4642) | 14.65  (680/4642) |
| *Category* | *No* | *I don't know* | *For public interest* | *For scientific research* | *For extra services or individual service* | *I would be paid for it* |
| Managerial position / Senior | 31.74  (432/1361) | 6.89  (94/1361) | 12.47  (170/1361) | 24.55  (334/1361) | 10.73  (146/1361) | 13.62  (185/1361) |
| Junior white collar | 31.77  (280/880) | 10.86  (96/880) | 11.10  (98/880) | 24.09  (212/880) | 8.10  (71/880) | 14.08  (124/880) |
| Worker | 27.19  (588/2162) | 14.52  (314/2162) | 14.03  (303/2162) | 22.70  (491/2162) | 7.47  (162/2162) | 14.09  (305/2162) |
| Self-employed or sole trader | 30.73  (148/480) | 10.88  (52/480) | 8.69  (42/480) | 22.79  (109/480) | 11.61  (56/480) | 15.31  (73/480) |
| At school or student | 26.21  (126/480) | 9.12  (44/480) | 9.35  (45/480) | 32.30  (155/480) | 7.78  (37/480) | 15.25  (73/480) |
| Pensioner | 39.17  (376/960) | 11.95  (115/960) | 11.62  (112/960) | 23.13  (222/960) | 2.48  (24/960) | 11.63  (112/960) |
| ^b^Other | 28.14  (405/1441) | 18.29  (264/1441) | 12.09  (174/1441) | 18.48  (266/1441) | 6.95  (100/1441) | 16.04  (231/1441) |

^a^Other education: corresponds to vocational education, matriculation or other types of education

^b^Other: corresponds to other types of jobs or status such as at-home mother/father
